# Parathyroid hormone-related peptide and parathyroid hormone-related peptide receptor type 1 in locally advanced laryngeal cancer as prognostic indicators of relapse and survival

**DOI:** 10.1186/s12885-022-09748-1

**Published:** 2022-06-27

**Authors:** Giovanni Almadori, Antonella Coli, Eugenio De Corso, Dario Antonio Mele, Stefano Settimi, Giovanni Di Cintio, Francesca Brigato, Domenico Scannone, Libero Lauriola, Franco Oreste Ranelletti

**Affiliations:** 1 Unit of Head and Neck Surgical Oncology, Fondazione Policlinico Universitario A. Gemelli IRCCS, Largo A. Gemelli 8, 00168 Rome, Italy; 2 Division of Pathological Anatomy, Fondazione Policlinico Universitario A. Gemelli IRCCS, Roma-Università Cattolica del Sacro Cuore, Rome, Italy; 3 Unit of Otorhinolaryngology – Head and Neck Surgery, Fondazione Policlinico Universitario A. Gemelli IRCCS, Rome, Italy; 4 Formerly Institute of Histology, Università Cattolica del Sacro Cuore, Rome, Italy

**Keywords:** Laryngeal cancer, Bio-radiotherapy with cetuximab, PTHrP and PTH1R, HER1, Prognostic and predictive role

## Abstract

**Background:**

Parathyroid hormone-related peptide (PTHrP) overexpression and poor patient outcome have been reported for many human tumors, but no studies are available in laryngeal cancer. Therefore, we studied the expression of PTHrP and its receptor, parathyroid hormone-related peptide receptor type 1 (PTH1R), in primary locally advanced laryngeal squamous cell carcinomas (LALSCC) also in relation to the clinical outcome of patients.

**Methods:**

We conducted a retrospective exploratory study, using immunohistochemistry, on PTHrP, PTH1R and HER1 expressions in LALSCC of 66 patients treated with bio-radiotherapy with cetuximab.

**Results:**

The expressions of PTHrP and PTH1R in LALSCC were associated with the degree of tumor differentiation (*p* = 0.01 and 0.04, respectively). Poorly differentiated tumors, with worse prognosis, expressed PTHrP at nuclear level and were PTH1R negative. PTHrP and PTH1R were expressed at cytoplasmic level in normal larynx epithelium and more differentiated laryngeal cancer cells, suggesting an autocrine/paracrine role of PTHrP in squamous cell differentiation of well differentiated tumors with good prognosis. Eighty-one percent HER1 positive tumors expressed PTHrP (*p* < 0.0001), mainly at nuclear level, consistent with the known up-regulation of *PTHrP* gene by HER1 signaling. In multivariable analyses, patients with PTHrP positive tumors had a higher relative risk of relapse (HR = 5.49; CI 95% = 1.62–22.24; *p* = 0.006) and survival (HR = 8.21; CI 95% = 1.19–105.00; *p* = 0.031) while those with PTH1R positive tumors showed a lower relative risk of relapse (HR = 0.18; CI 95% = 0.04–0.62; *p* = 0.002) and survival (HR = 0.18; CI 95% = 0.04–0.91; *p* = 0.029).

**Conclusions:**

In LALSCC nuclear PTHrP and absence of PTH1R expressions could be useful in predicting response and/or resistance to cetuximab in combined therapies, contributing to an aggressive behavior of tumor cells downstream to HER1.

**Supplementary Information:**

The online version contains supplementary material available at 10.1186/s12885-022-09748-1.

## Introduction

Laryngeal squamous cell carcinoma remains one of the most common cancer of the respiratory tract. Although the overall incidence is declining and despite advances in multimodal therapy, the overall 5-year survival rate of patients with locally advanced laryngeal squamous cell carcinoma (LALSCC) remains poor, with a 5-year survival rate ranging from 66 to 63%, over the past 40 years [1].

Non–surgical organ preservation strategies led to a shift from total laryngectomy to combined platinum-based chemo-radiotherapy (CRT) or neoadjuvant cisplatin and fluorouracil followed by radiotherapy (RT) or RT alone, with beneficial effects on many patients, although functional complications and decreased overall survival have been observed in older patients [2–4].

Parathyroid hormone-related peptide (PTHrP) is involved in cell growth and differentiation [5] and it is expressed in three isoforms of 139, 141, and 173 amino acids. The post-translational processing of these proteins leads to a family of peptides with paracrine, autocrine, or intracrine functions [6]. These functions depend upon cell type expressing PTHrP and the NH_2_-terminal region (PTHrP 1–34), mid-molecule (PTHrP 38–94 or 38–102) or COOH-terminal region (PTHrP 109–141) involved in the signaling process [7–10]. The amino-terminal region of PTHrP (1–34) presents similarities to that of parathyroid hormone, including the PTH/PTHrP type 1 receptor (PTH1R), which is expressed at the plasma membrane of cell lines and tissues [11]. In vitro and in vivo evidences indicate that PTHrP is involved in tumor initiation, growth and metastatic spread [12–14]. Finally, the overexpression of PTHrP is associated with poorly differentiated oral squamous cell carcinoma, characterized by poor overall survival [15].

Epidermal growth factor receptor (HER1) overexpression is a frequent molecular alteration in head and neck squamous cell carcinoma (HNSCC) and its increased expression and activity have been associated with worse clinical outcome and resistance to RT [16–18]. In this respect, the anti-HER1 monoclonal antibody cetuximab was approved by US Food and Drug Administration, in addition to radiotherapy, for the definitive treatment of resectable LALSCC [19] and it has been widely used in the first-line recurrent and/or metastatic settings, improving overall survival when combined with chemotherapy [20, 21]. However, only a minority of patients in these settings benefited from cetuximab treatment, due to the occurrence of intrinsic and/or acquired resistance to therapy [22]. Thus, it is critical to identify predictive biomarkers of sensitivity/resistance to cetuximab in HNSCC.

Several authors indicated that *PTHrP* gene expression is regulated by HER1 signaling in a variety of normal and cancer cell types [14, 23–27], thus contributing to the malignant behavior of tumor cells downstream of HER1 signaling. For example, in oral squamous cell carcinoma, PTHrP gene expression is up-regulated by HER1 signaling through the MAPK cascades and leads to enhanced cell proliferation, migration and invasion [28]. PTHrP overexpression, at mRNA level, was reported in six squamous cell carcinomas of the larynx [29]. However, to our knowledge, there are no studies correlating the expression of both PTHrP and PTH1R in human squamous carcinoma of the larynx, with the clinical-pathological characteristics of patients.

Therefore, we planned to study the expression of PTHrP and its receptor, PTH1R, in LALSCC also in relation to the clinical outcome of patients. Furthermore, since PTHrP is up-regulated by HER1 and contributes to tumor malignancy downstream to HER1, we investigated if the expression of PTHrP and PTH1R could be prognostic and/or predictive biomarkers of sensitivity/resistance to bio-radiotherapy with cetuximab.

## Methods

### Patients and study design

This study is a retrospective analysis on a cohort of patients admitted to our Department of Otorhinolaryngology-Head and Neck Oncology, between 1999 and 2005 (Institutional Review Tumor Board “SpiderNet”). We utilized biological samples and data collected at our Head and Neck Cancer Center during the participation at the randomized phase III trial comparing radiation therapy alone with radiation therapy plus concomitant cetuximab, for locally advanced squamous cell carcinoma of the head and neck [30]. Eligible patients had a histologically confirmed squamous cell carcinoma, no distant metastases and no prior therapy. Due to heterogeneity in clinical behavior of larynx cancer relatively to anatomical subsites, we excluded all patients with primary supraglottic LSCC. At that time, all patients were initially evaluated with a comprehensive head and neck examination and then staged and discussed by the multidisciplinary tumor board. Histopathological grading was assessed according to WHO guidelines [31]. We included in this study 66 subjects treated with bio-radiotherapy with cetuximab and concurrent intensity-modulated radiotherapy (IMRT), if they were considered suitable for an organ preservation protocol according to international guidelines, or in case of cT4 disease and any N, if they refused total laryngectomy (TL). In case of histologically proven persistent or recurrent loco-regional disease, salvage TL, with or without neck node dissection, was performed. Cetuximab was administered at an initial dose of 400 mg/m2 during the week before IMRT and then 250 mg/m2 per week during IMRT with a maximum of 7 additional doses. We delivered 69.96 Gy in 33 fractions to the planning target volume (PTV) including the gross tumor volume; 59.4 Gy in 33 fractions to the PTV of the high-risk clinical target volume (CTV), and 54 Gy in 33 fractions to the PTV of the low-risk CTV. Clinically negative neck regions were treated with a dose of 50 to 54 Gy, while gross nodal disease received full-dose radiotherapy (70–76.8 Gy), depending on fractionation. All patients underwent regular early follow-up visit at 4 weeks and 8 weeks after completion of radiotherapy. During the first and second years, they were therefore evaluated every 3 months; subsequently, every 6 months up to 5th year of follow-up.

### Immunohistochemistry

For immunohistochemistry, consecutive 4 μm tissue sections of representative paraffin blocks from each case were processed as previously reported [32]. Briefly, slides were immunostained on a Dako autostainer (Dako), using the Vectastain ABC peroxidase kit (Vector Laboratories; Burlingame, CA). Sections were incubated with rabbit polyclonal antibody against PTHrP 1–34 (1:250; D.B.A.; Milano, Italy), mouse anti-human monoclonal antibodies against PTH1R (1:100; clone 3D1.1; Santa Cruz Biotechnology, Inc.; Santa Cruz, CA) and anti-HER1 (clone H11, dilution 1:150; Dako, Milano, Italy). In order to evaluate the proliferative activity of laryngeal cancers in relation to the expression of PTHrP, data relative to minichromosome maintenance protein 7 (MCM7) labeling index were derived from our previous study on the same cohort of patients [33].

In order to achieve the mean percentage of immunostained cancer cells in each single case, sections were extensively examined by three independent observers (AC, LL, FOR). We choose to score the percentage of the immunostained cells from grade 1 to 3, as follows: 1: < 30%; 2: > 30 - < 50%; 3: > 50%, of tumor cells. For statistical analyses, grade 1 and grades 2 + 3 were considered as low and high expressing tumors, respectively. In each single case, the section was extensively examined and the cytoplasmic and/or nuclear PTHrP immunostaining was recorded together with tumor differentiation pattern**.** As the intensity of immunolabeling could vary between different analysis sessions, only the percentage of clearly labeled cells was used for the purpose of positivity definition. Disagreements (< 5%) were reviewed followed by conclusive consensus.

### Statistical analysis

The primary endpoints went from the date of the beginning of the bio-radiotherapy with cetuximab to the date of clinical or pathological loco-regional or distant recurrence (relapse-free survival: RFS) or to the date of death, regardless of the cause (overall survival: OS) or to the date of the last available information on the patient’s status.

All medians and life tables were computed using the product-limit estimate by Kaplan and Meier, and the curves were examined by means of the log-rank test. Univariable and multivariable analyses were performed by Cox’s proportional hazards model. The proportional hazards assumption was assessed by visual inspection of log-log survival curves and linear regressions of scaled Schoenfeld residuals versus time. Collinearity was verified by computing variance inflation factors from the covariance matrix of parameter estimates. Relapse-free and overall survival probabilities, given the covariates and follow-up time, were calculated for the model fitted by the multivariable Cox regression. Kruskall-Wallis tests were used to analyse the distribution of PTHrP-PTH1R phenotypes according to clinico-pathological parameters. Numerically stable version of fast backward elimination on factors was utilized to obtain a simplified model that can predict the predicted values of the full model with good accuracy. This method uses the fitted complete model and computes approximate Wald statistics by computing conditional (restricted) maximum likelihood estimates assuming multivariate normality of estimates [34, 35]. The performance of the final Cox models was assessed with respect to calibration and discrimination. Calibration was examined using calibration curves of the relationship between the observed survival rate and the predicted probabilities of relapse-free and overall-survival. Overfitting-corrected estimates of the performance of the final Cox models were evaluated by bootstrap with resampling with 200 repetitions, using adaptive linear spline hazard regression [36] and estimating the absolute mean error. Discrimination was evaluated by the concordance index (C-index) as the final Cox model ability to separate patient’s outcomes [35]. Two sided *p* < 0.05 was considered significant in statistical tests. Analyses were performed using the JMP version 13.2 (SAS Institute Inc., Cary, NC, USA) and RStudio software version 3.3.3 (R Development Core Team: A language and environment for statistical computing. R Foundation for Statistical Computing Vienna, Austria, 2011).

## Results

### PTHrP and PTH1R expression

PTHrP low and high expressing tumors were 26/66 (39.4%) and 40/66 (60.6%), respectively. PTH1R low and high expressing tumors were 38/66 (57.6%) and 28/66 (42.4%), respectively (Table 1).

**Table 1:** Parathyroid hormone-related peptide and parathyroid hormone-related peptide type 1 receptor immunostaining according to clinicopathologic characteristics in 66 patients with advanced laryngeal squamous cell carcinoma

		PTHrP			PTH1R		
	N	Negative	Positive	p^a^	Negative	Positive	p
Gender:							
Male	61	23 (38%)	38 (62%)		35 (57%)	26 (43%)	
Female	5	3 (60%)	2 (40%)	0.37	3 (60%)	2 (40%)	1.0
Age (years):							
< 60	26	4 (24%)	13 (76%)		13 (76%)	4 (24%)	
> 60	40	22 (45%)	27 (55%)	0.15	25 (51%)	24 (49%)	0.09
Site:							
Glottic	47	22 (47%)	25 (53%)		27 (57%)	20 (43%)	
Transglottic	19	4 (21%)	15 (79%)	0.09	11 (58%)	8 (42%)	1.0
Differentiation:							
Well-Moderately	32	18 (56%)	14 (44%)		14 (44%)	18 (56%)	
Poorly	34	8 (24%)	26 (76%)	**0.01**	24 (71%)	10 (29%)	**0.04**
T classification:							
T2	36	13 (36%)	23 (64%)		20 (56%)	16 (44%)	
T3-4	30	13 (43%)	17 (57%)	0.62	18 (60%)	12 (40%)	0.80
Stage:							
II	29	10 (34%)	19 (66%)		15 (52%)	14 (48%)	
III-IV	37	16 (43%)	21 (57%)	0.61	23 (62%)	14 (38%)	0.46
Lymph-node:							
Negative	50	21 (42%)	29 (58%)		29 (58%)	21 (42%)	
Positive	16	5 (31%)	11 (69%)	0.56	9 (56%)	7 (44%)	1.0
HER1							
Negative	24	18 (75%)	6 (25%)		12 (50%)	12 (50%)	
Positive	42	8 (19%)	34 (81%)	**<0.0001**	26 (62%)	16 (38%)	0.44

^a^ Two-tail Fisher’s exact test

**Fig. 1:**
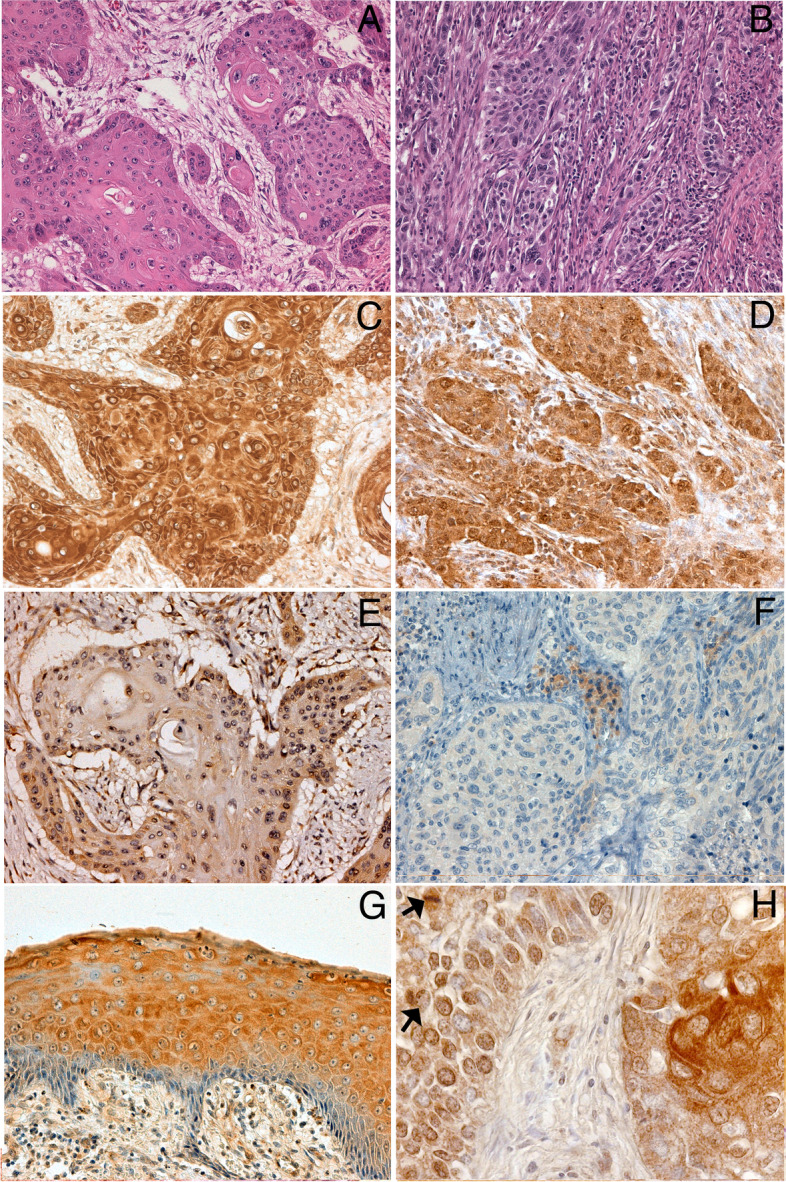
Immunohistochemical analysis of PTHrP (**C**, **D**, **G**, **H**) and PTH1R (**E**, **F**) in a well differentiated (**A**, **C**, **E**, **G**) and a poorly differentiated (**B**, **D**, **F**, **H**) laryngeal squamous cell carcinoma. (H&E in **A** and **B** top boxes). Immunostainings of PTHrP in normal epithelium close to the relative tumor areas (**G**). In H: PTHrP cytoplasmic immunostaining of cancer cells in more differentiated tumor area (on the right side) and PTHrP nuclear immunostaining of poorly differentiated cancer cells (on the left side); arrows indicate two cancer cells in mitosis. (original magnifications: **A**-**G**: × 200, **H**: × 400)

The analysis of data regarding the expressions of PTHrP and PTH1R showed an association with the degree of tumor differentiation. In fact, PTHrP was more frequently expressed in poorly differentiated tumors (Fisher’s exact test: *p* = 0.01), prevalently in absence of PTH1R expression. On the other hand, PTH1R was more frequently expressed in differentiated tumors, with statistical significance (Fisher’s exact test: *p* = 0.04). Moreover, 34/40 (85%) PTHrP expressing tumors were also HER1 positive (Fisher’s exact test: *p* < 0.0001), while the expression of PTH1R was not significantly associated with that of HER1 (Fisher’s exact test: *p* = 0.44) (Table 1). PTHrP was expressed in the tumor cells at cytoplasmic and/or nuclear levels (Fig. 1). In particular, PTHrP was expressed at nuclear level in 88.9% of poorly differentiated tumors and in 11.1% of well differentiated ones (Fisher’s exact test: *p* = 0.025; Fig. 1). Moreover, tumors expressing PTHrP at nuclear level showed an higher MCM7 labeling index than tumors not expressing nuclear PTHrP (mean labeling index ± S.E: 74.0 ± 5.54, *N* = 10 versus 56.5 ± 3.0, *N* = 34; Mann-Whitney test: *p* = 0.018). In normal squamous epithelium, PTHrP was expressed only in the cytoplasm of suprabasal differentiating cells (Fig. 1G). At the tissue level, the tumor cells in the most differentiated areas of the tumor expressed PTHrP at the cytoplasmic level while those poorly differentiated in the proliferating areas expressed PTHrP at the nuclear level (Fig. 1H).

Considering PTHrP-PTH1R co-expression status, PTHrP high expression was more frequent in poorly differentiated tumors not expressing PTH1R. On the contrary, all well differentiated, PTHrP positive tumors co-expressed PTH1R (Table 2 and Fig. 1).

**Table 2:** PTHrP-PTH1R expression status according to tumor differentiation

		Tumor Differentiation		
Tumor phenotype:	N (% of total)	Well N (%)	Moderately N (%)	Poorly N (%)
PTHrP-PTH1R-	15 (22.7%)	5 (33.3%)	5 (33.3%)	5 (33.3%)
PTHrP-PTH1R+	11 (16.7%)	3 (27.3%)	5 (45.4%)	3 (27.3%)
PTHrP+PTH1R-	23 (34.8%)	0	4 (17.4%)	19 (82.6%)
PTHrP+PTH1R+	17 (25.8%)	6 (35.3%)	4 (23.5%)	7 (41.2%)
Total	66	14 (21.2%)	18 (27.3%)	34 (51.5%)

### Survival analysis

During the follow-up period (range: 2–139 months; median time: 32; C.I.95%: 27–45), loco-regional recurrences were observed in 26 of 66 (39.4%) cases. At the end of the study, 14 of 66 (21.2%) patients were dead of cancer.

**Fig. 2:**
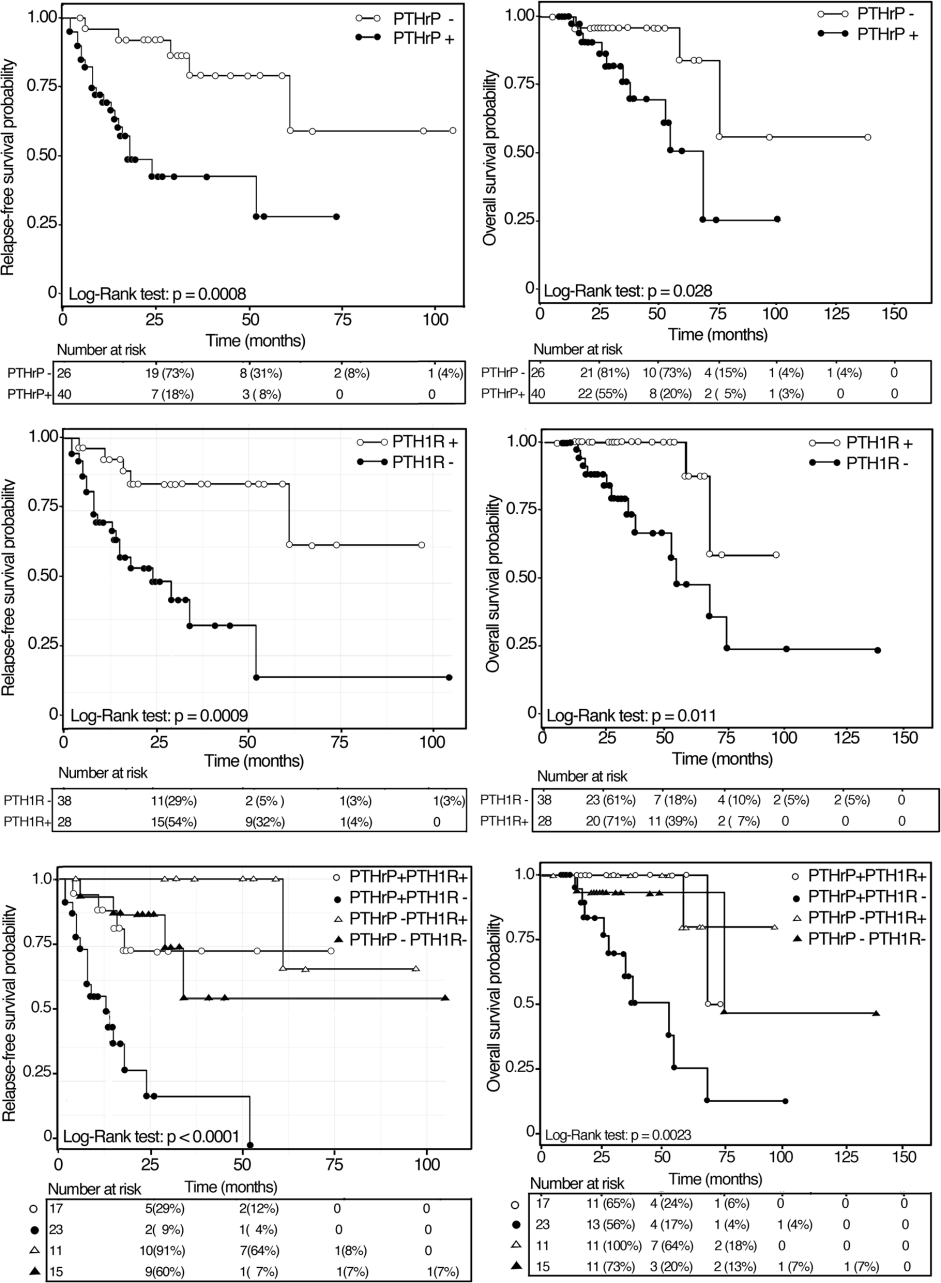
Kaplan-Meier analyses of relapse-free and overall survival curves of 66 laryngeal squamous cell carcinoma patients as a function of positive and negative PTHrP and PTH1R expressions and of PTHrP-PTH1R phenotypes of their tumors

A significant relationship was found between tumor PTHrP and PTH1R expression and patient survival (Fig. 2). Kaplan-Meier analysis of survival curves revealed that after 5-year follow-up the estimated relapse-free survival was 79.4% ± 9.6 S.E. and 28.9% ± 13.4 S.E. for patients with PTHrP negative and positive tumors, respectively. On the contrary, the estimated relapse-free survival was 17.7% ± 10.0 S.E. and 64.9 ± 16.7 S.E. for patients with PTH1R negative and positive tumors, respectively. Similarly, patients with PTHrP positive and negative tumors showed an estimated overall surviving of 50.8% ± 13.7 S.E. and 96.0% ± 3.9 S.E., respectively, while those with PTH1R positive and negative tumors showed an estimated overall surviving of 87.5% ± 11.7 S.E. and 47.6% ± 13.6 S.E., respectively. Kaplan-Meier analysis of survival curves of patients grouped by PTHrP-PTH1R co-expression status of their tumors, revealed that the co-expression of PTH1R significantly prolonged both relapse-free and overall survival times (Fig. 2).

Table 3 lists the results of univariable analysis of prognostic variables for relapse-free and overall survival. Patients with poorly differentiated tumors, transglottic tumor site, PTHrP positive and PTH1R negative tumors showed a significantly increased risk of relapse. Apart from tumor site, the same is true for the risk of death (Table 3).

**Table 3:** Univariable analysis of relapse-free and overall survival in 66 laryngeal squamous cancer patients

Variables:		Relapse-free survival			Overall survival		
	N	RR^a^	C.I. 95%	p^b^	RR	C.I. 95%	p
Age (risk per year)	66	0.99	0.9-1.0	0.80	0.98	0.9-1.1	0.23
Gender							
Female	5	1			1		
Male	61	1.60	0.3-28.8	0.62	0.81	0.2-14.8	0.84
Differentiation							
Well-Moderately	32	1			1		
Poorly	34	5.62	2.3-15.6	0.0001	2.82	0.9-9.3	0.06
Site							
Glottic	47	1			1		
Transglottic	19	2.57	1.1-5.7	0.028	1.15	0.2-3.7	0.84
T							
2	36	1			1		
3-4	30	1.64	0.7-3.7	0.22	2.31	0.8-7.7	0.13
Stage							
II	29	1			1		
III-IV	37	1.19	0.5-2.7	0.70	2.46	0.8-10.9	0.14
Lymph nodes							
Negative	50	1			1		
Positive	16	0.97	0.4-2.8	0.95	1.28	0.3-3.9	0.69
PTHrP							
Negative	37	1			1		
Positive	29	4.35	1.7-13.1	0.0011	3.84	1.2-17.3	0.02
PTH1R							
Positive	19	1			1		
Negative	47	3.11	1.3-8.5	0.0085	5.64	1.5-36.5	0.0076

^a^Reference risk; ^b^Likelihood ratio test

**Fig. 3:**
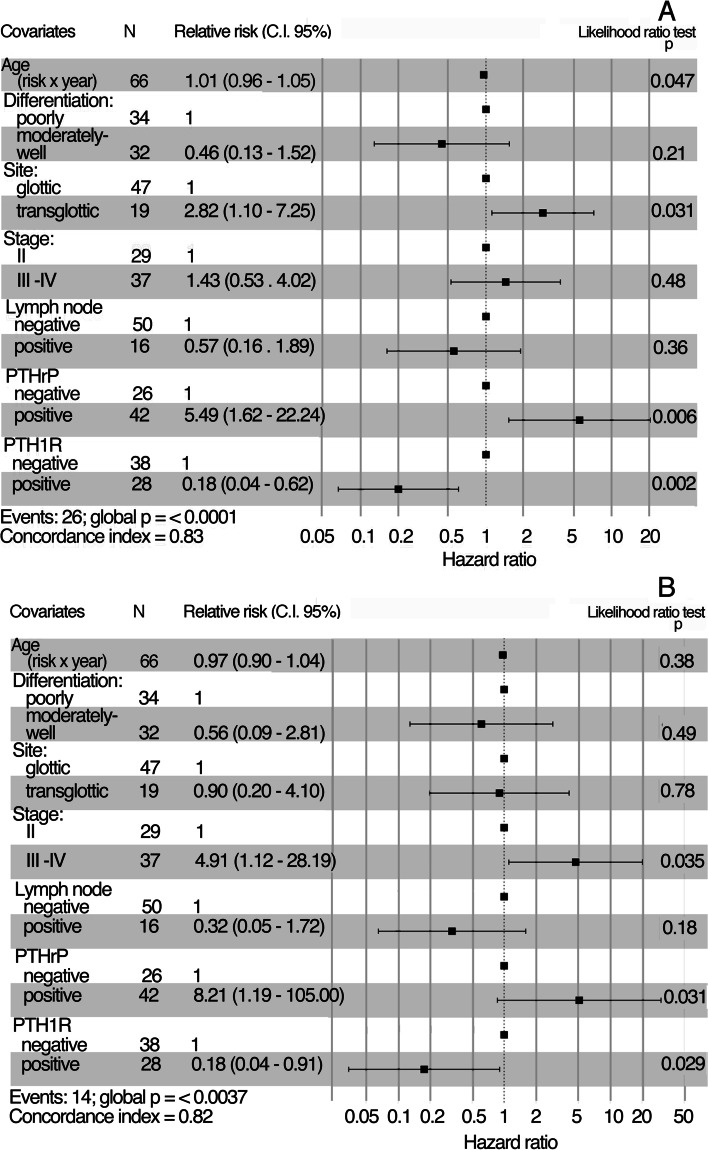
Multivariable analyses of prognostic variables for relapse-free (**A**) and overall (**B**) survival. Forest plot of the relative estimates

In multivariable Cox regression analysis, increasing age, transglottic tumor site, PTHrP positive and PTH1R negative tumors retained an independent prognostic significance of increased risk of recurrence, while, high III-IV stage and PTHrP positive and PTH1R negative tumors behaved as independent prognostic indicators of increased risk of death (Fig. 3).

**Fig. 4:**
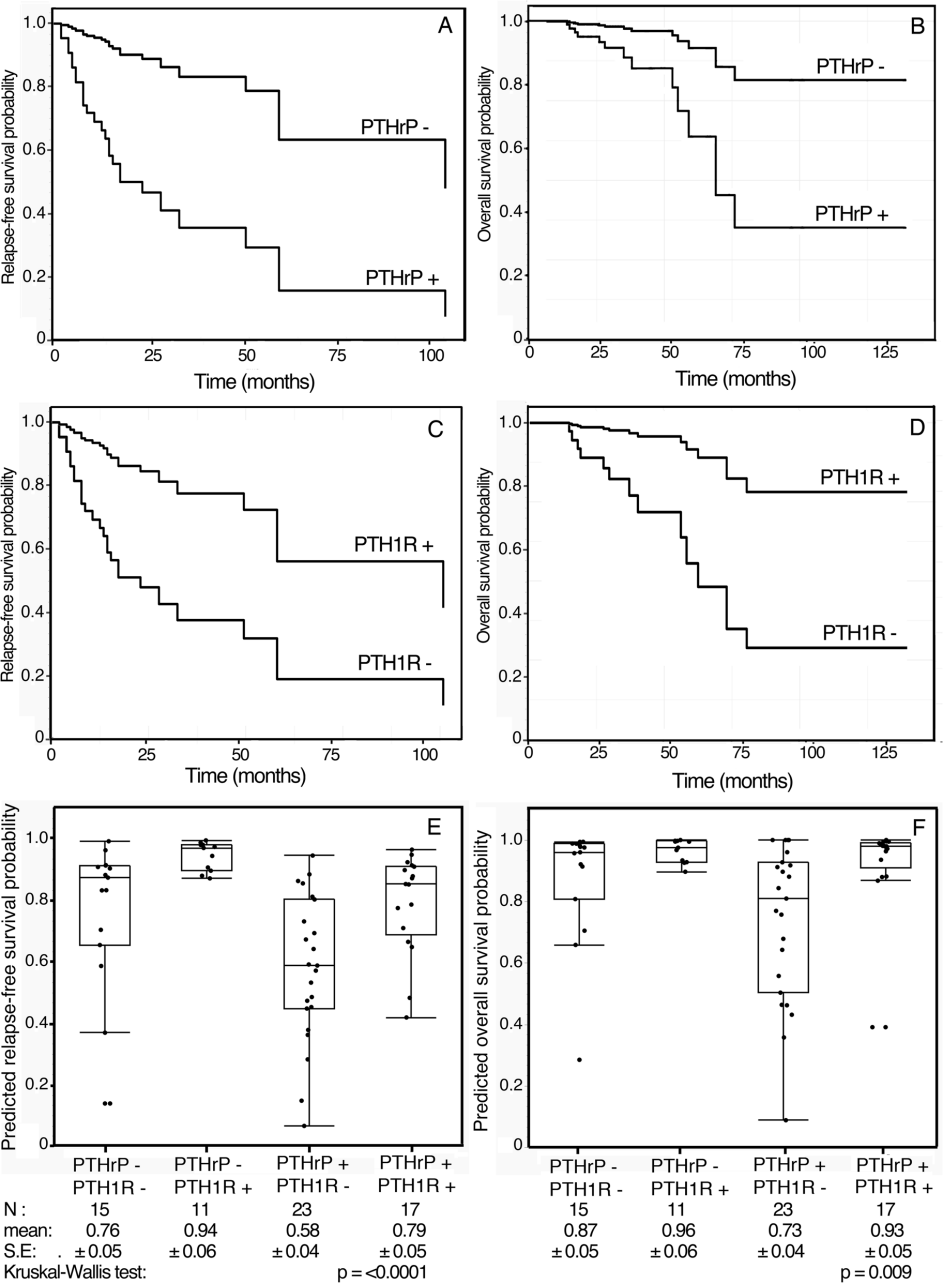
Relapse-free and overall survival curves adjusted for confounding variables based on the multivariable Cox proportional hazards model. PTHrP (**A**, **B**) and PTH1R (**C**, **D**). Plots of the relapse-free (**E**) and overall (**F**) survival probabilities predicted by the multivariable Cox’s regression fitted model as a function of PTHrP-PTH1R phenothypes of laryngeal squamous cell carcinomas

Adjusted survival curves, calculated based on Cox model, show the decreased relapse-free and overall survival rates for patients bearing PTHrP positive and PTH1R negative tumors (Fig. 4). Moreover, the mean expected survival probability of patients with PTHrP positive and PTH1R negative tumor phenotype was significantly lower than that of patients with both PTHrP and PTH1R positive tumor phenotype (Fig. 4).

Simplified models were obtained from the fitted complete model by means of backward elimination on factors, considering as covariate PTHrP-PTH1R status of the tumor. The factors remaining in the models were age at presentation, tumor site and PTHrP-PTH1R expression status, relative to relapse, and stage and PTHrP-PTH1R expression status, relative to survival. The results of Cox’s proportional hazard analysis are reported in Supplementary Table 1. These models were internally validated with respect to discrimination and calibration. Discrimination suggested a good accuracy with a optimism-corrected C-index of 0.81 for RFS and OS. The closeness of the calibration curves for RFS and OS to the ideal 45° calibration lines suggests that the models are well-calibrated for predictions on an absolute probability scale (Supplementary Fig. 1). The absolute values of the differences between the predicted and the observed values (Mean Absolute Error: MAE) were 0.042 and 0.032 for RFS at 24 and 36 months, and 0.027 and 0.008 for OS at 36 and 60 months, respectively. Moreover, the optimism-corrected slope shrinkages of 0.81 for RFS and OS suggests little overfitting.

## Discussion

LALSCC continues to represent a therapeutic challenge. Then, a better understanding of the tumor phenotypes and molecular characteristics associated with a poor outcome could help to select patients for most appropriate targeted treatments.

Since many clinical data support a pro-tumorigenic role of PTHrP by modulating proliferation, apoptosis and cell survival and acting as a negative regulator of tumor cell dormancy [37], we investigated in LALSCC the contribution of the expression pattern of PTHrP and PTH1R to tumor behavior and clinical outcome.

Accumulating in vitro and in vivo evidences indicate that the secreted and nuclear forms of PTHrP have distinct effects on cellular functions. For instance, in cultured vascular smooth muscle cells, nuclear targeting of PTHrP is associated with a striking increase in mitogenesis, while an opposite effect results from interaction of PTHrP with cell surface receptors [9]. In breast, prostate, and colon cancer cells the PTHrP intranuclear pathway stimulates cell proliferation, protects cells from apoptosis or anoikis, and enhances cell migration, whereas secreted PTHrP inhibits cell proliferation and promotes cell death [38–41].

In agreement with previous observations in several tumors, such as breast, colon, prostate, gastric and oral squamous cancers [22, 42–45], we found that an high expression of PTHrP is more frequently observed in poorly differentiated laryngeal cancers and is associated with an increased risk of relapse and death. Interestingly, we observed that in poorly differentiated laryngeal tumors PTHrP was expressed mainly at nuclear level, suggesting an intracrine action in these aggressive histological types.

As assessed by MCM7 labeling index, the laryngeal cancers expressing PTHrP at nuclear level showed a higher proliferative activity than those expressing PTHrP in the cytoplasm. Moreover, also at the tissue level, PTHrP was expressed in the cytoplasm of the cells in the most differentiated tumor areas and in the nucleus of the poorly differentiated cells in the proliferating tumor areas.

In normal human skin epithelium, PTHrP localizes in the cytoplasm of the keratinized cell layer [46]. Interestingly, we observed that PTHrP and PTH1R were present in the cytoplasm of the more differentiating cells of the laryngeal squamous cancer in addition to the keratinized cells of supra-basal layers of the normal adjacent larynx epithelium. Noteworthy, it has been previously demonstrated that the PTHrP role in inhibiting growth and supporting differentiation of keratinocytes in culture is, at least in part, mediated through PTH1R action [47]. In fact, as revealed by the survival analyses, the expected survival probability of patients with laryngeal cancer expressing PTHrP seems correlated with co-expression of PTH1R. Considering that poorly differentiated tumors in the majority of cases express nuclear PTHrP and are PTH1R negative, we suggest an intracrine role of PTHrP in more aggressive laryngeal cancers, whereas a PTH1R-dependent, paracrine role of PTHrP mainly characterizes primary tumors with a good prognosis.

Recently, in neuroblastoma it has been reported that the oncogenic role of PTHrP is a consequence of its intracrine function, as downregulation of its receptor, PTH1R, increased anchorage independent growth and induced a more undifferentiated, invasive phenotype [14]. Interestingly, in oral cancer, in contrast to what we observed in laryngeal cancer, Chang et al. recently reported that PTHrP promotes cell growth via an autocrine/paracrine pathway [27]. However, it is difficult to compare our results with those reported by Chang et al. [27], since in their study the roles of PTH1R and nuclear PTHrP expression on tumor behavior are not specified. On the other hand, it is also to be considered that head and neck squamous cell carcinomas show considerable heterogeneity in clinical behavior [48]. In this regard, it is noteworthy that different tissues or malignancies have specific and different post-translational processing, producing different proportions of daughter peptides from the three PTHrP isoforms, leading to prevalent paracrine, autocrine, or intracrine effects [6, 49]. In fact, in lung carcinoma, the expression of amino and carboxyl regions of PTHrP can vary independently in different tumor cases, so that the presence of amino PTHrP and PTH1R worsen the prognosis in lung cancer patients [32], while, carboxyl PTHrP, when present, may lower the stimulatory effect of amino PTHrP [50]. Moreover, many evidences indicate that discrete PTHrP fragments exert multiple effects on different neoplastic cell populations via cytosolic and nuclear targets, by functioning in different settings either as inducers of cell proliferation or leading to cell death and apoptosis [42]. As a matter of fact, in oral cancer PTHrP regulates the cell motility and invasiveness via an autocrine and an intracrine pathway, respectively [28], but in osteosarcoma, loss of PTH1R induced a decrease in proliferation and an increase in tumor differentiation, not mediated by extracellular PTHrP and likely induced by an intracrine PTHrP pathway [51].

Our results in larynx cancer, show the variability of PTHrP and PTH1R expression (nuclear and/or cytoplasmic), emphasizing the relevance of a careful evaluation of immunohistochemical phenotypes besides tumor histology, in order to draw useful information concerning tumor characteristics and patient survival. Considering that in poorly differentiated, more aggressive tumors PTHrP is mainly localized at the nuclear level and PTH1R is rarely expressed, it is possible to hypothesize that the effect of nuclear targeting PTHrP on proliferation may override the receptor-mediated inhibition of proliferation. Moreover, in the present study, we found that HER1 and PTHrP were coexpressed, particularly in poorly differentiated laryngeal tumors, a finding not surprising, considering that HER1 signaling regulates *PTHrP* gene expression in a variety of normal and cancer cell types [14, 23–27]. Likewise, in oral cancer cells in culture, PTHrP was significantly up-regulated by EGF stimulation via ERK and p38 MAPK, contributing to the malignancy of tumor cells downstream of HER1 signaling [28].

To date, no predictive biomarkers for effectiveness anti-HER1 treatment in HNSCC are available. Therapeutic resistance to anti-HER1 therapy may arise from alternative pathways overcoming the reduced HER1 signaling and/or modulating the HER1-dependent signaling [52, 53]. In particular, overexpression of HER1, as assessed by immunohistochemistry, could not be correlated with the level of response to cetuximab treatment [54].

In our cohort of LASCC patients treated with bio-radiotherapy with cetuximab, expression of nuclear PTHrP seems to be a prognostic/predictive marker of relapse and poor survival, when expressed in the absence of PTH1R. Therefore, nuclear PTHrP expression could be useful in predicting resistance to cetuximab-combined treatment in laryngeal cancers, by contributing to an aggressive behavior of tumor cells downstream to HER1. This possibility seems to be supported by the observation, in oral cancer cells, that combined treatment with an HER1 inhibitor (AG1478) and PTHrP knockdown achieved a synergistic inhibition of malignant phenotypes, more effective than each single treatment. Moreover, PTHrP silencing inhibited MAPK and phosphatidylinositol 3-kinase (PI3K)/Akt signaling, which are involved in cell proliferation, survival, and motility [28].

Many evidences indicate that PI3K plays a key role in the progression of HNSCC and development of resistance to cetuximab [55]. In particular, the inhibition of Ras and PI3K signaling might be effective to overcome cetuximab-resistance and radioresistance in HNSCC tumor cells [56]. Interestingly, in colon and prostate cancer cells, nuclear PTHrP exerts intracrine inhibitory effects on apoptosis via activation of the PI3K/Akt pathway through integrin α6β4 induction. In addition, PTHrP affects the phosphorylation state of Akt substrates implicated in apoptosis suppression [57, 58].

In the light of the above mentioned literature data, it can be suggest that, also in laryngeal cancer, the intracrine effect of nuclear PTHrP can play a role in the tumor progression and development of resistance to cetuximab-combined treatment, through the activation of the PI3K/Akt pathway. In support of this argument, PTHrP targeting resulted in prolonged survival of hypercalcemic mice, as well as decreased cell proliferation of renal carcinoma and increased apoptosis of human medulloblastoma’s cell lines [59–61].

This study has some limitations: this model was derived from retrospective data, making it susceptible to a data collection bias and the portability to other patient cohorts of this model needs to be externally validated, although the optimism-corrected slope shrinkage indicates little over fitting. However, a most suitable design of future retrospective and/or prospective studies in order to overcome these hindrances is needed.

## Conclusions

In conclusion, this study analyzes for the first time the patterns of PTHrP and PTH1R expression in LALSCC in relation to tumor histology and grade. In this exploratory study we developed an internally validated simple prognostic model based on the patterns of PTHrP and PTH1R expression that allows to improve the prognostic stratification of patients with HER1 positive LALSCC that could benefit from bioradiotherapy with cetuximab. This can be of relevance in order to achieve, particularly in this patient’s subset, a balance between overall survival, larynx preservation, and quality of life. Moreover, our results provide arguments in favor of the possible advantage of targeting PTHrP to reduce cetuximab-combined treatment resistance of squamous laryngeal cancer patients.

## Supplementary Information

**Additional file 1: Supplementary Table 1**.

**Additional file 2: Supplementary Fig. 1.** Plots of bootstrap estimates of calibration accuracy for the indicated month estimates from the Cox models, using adaptive linear spline hazard regression. The gray scale line is the line of identity of observed-predicted relationship, representing the ideal calibration curve; the smooth black curve is the apparent calibration estimated by linear spline hazard regression; the blue line is the bootstrap overfitting-corrected calibration curve estimated also by hazard regression. Shown are mean absolute calibration error (MAE) and 0.9 Quantile of calibration error. The absolute error is the absolute value of the difference between the predicted value and the observed value.

## Data Availability

The datasets generated during and analyzed during the current study are not publicly available because the consent of the patients allows the publication of the data but not the transmission of the same to third parties. They are available from the corresponding author on reasonable request.

## Abbreviations

CRT: Chemoradiotherapy
CTV: clinical target volume
HER1: Epidermal growth factor receptor
HNSCC: Head and neck squamous cell carcinoma
IMRT: Intensity-modulated radiotherapy
LALSCC: Locally advanced laryngeal squamous cell carcinomas
MCM7: Minichromosome maintenance protein 7
PTH1R: Parathyroid hormone-related peptide receptor type 1
PTHrP: Parathyroid hormone-related peptide
PTV: planning target volume
RFS: Relapse-free survival
RT: Radiotherapy
