# Supplementary material for: Parathyroid hormone-related peptide and parathyroid hormone-related peptide receptor type 1 in locally advanced laryngeal cancer as prognostic indicators of relapse and survival
Source: BMC Cancer. 2022 Jun 27;22:704. doi: 10.1186/s12885-022-09748-1 (PMC9235225; doi:10.1186/s12885-022-09748-1)
Supplement: Supplementary file 1 — Additional file 1: Supplementary Table 1. [file 12885_2022_9748_MOESM1_ESM.docx]

|  | Relapse-free Survival | | |
| --- | --- | --- | --- |
| Variables | Relative Risk. | C. I. 95% | p |
| AGE: |  |  |  |
| (risk per year) | 0.97 | 1.01-1.02 | 0.08 |
| SITE: |  |  |  |
| glottic  transglottic | 1  3.66 | 1.53 - 3.69 | 0.011 |
| PTHrP-PTH1R status: |  |  |  |
| PTHrP+ PTH1R+ | 1 |  |  |
| PTHrP+ PTH1R- | 8.64 | 3.02 – 31.33 | <0.0001 |
|  |  | C index = 0.82 | |
|  | Overall Survival | | |
| STAGE: |  |  |  |
| II | 1 |  |  |
| III-IV | 2.85 | 0.9 – 12.8 | 0.083 |
| PTHrP-PTH1R status: |  |  |  |
| PTHrP+ PTH1R+ | 1 |  |  |
| PTHrP+ PTH1R- | 7.28 | 1.4 – 134.6 | 0.0032 |
|  |  | C index = 0.82 | |

Supplemental Table 1. Multivariable Cox’s proportional hazard analysis of reduced models.
