# Supplementary figures and images for: Parathyroid hormone-related peptide and parathyroid hormone-related peptide receptor type 1 in locally advanced laryngeal cancer as prognostic indicators of relapse and survival
Source: BMC Cancer. 2022 Jun 27;22:704. doi: 10.1186/s12885-022-09748-1 (PMC9235225; doi:10.1186/s12885-022-09748-1)

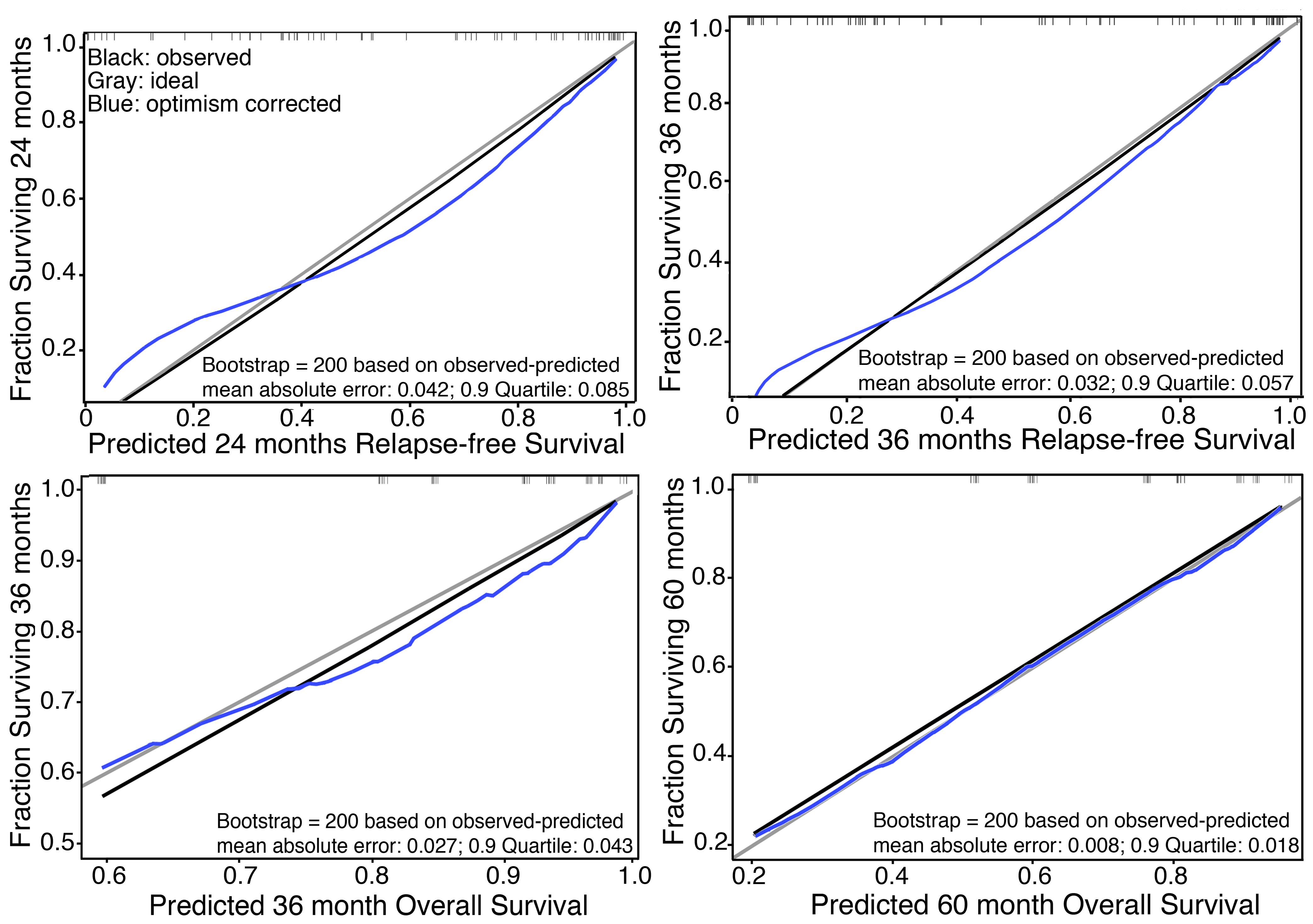

Supplement: Supplementary file 2 — Additional file 2: Supplementary Fig. 1. Plots of bootstrap estimates of calibration accuracy for the indicated month estimates from the Cox models, using adaptive linear spline hazard regression. The gray scale line is the line of identity of observed-predicted relationship, representing the ideal calibration curve; the smooth black curve is the apparent calibration estimated by linear spline hazard regression; the blue line is the bootstrap overfitting-corrected calibration curve estimated also by hazard regression. Shown are mean absolute calibration error (MAE) and 0.9 Quantile of calibration error. The absolute error is the absolute value of the difference between the predicted value and the observed value. [file 12885_2022_9748_MOESM2_ESM.tif]
